# Infrared reflectance imaging in age‐related macular degeneration

**DOI:** 10.1111/opo.12283

**Published:** 2016-04-25

**Authors:** Angelica Ly, Lisa Nivison‐Smith, Nagi Assaad, Michael Kalloniatis

**Affiliations:** ^1^ Centre for Eye HealthSydneyAustralia; ^2^ School of Optometry and Vision ScienceUniversity of New South WalesSydneyAustralia; ^3^ Department of OphthalmologyPrince of Wales HospitalRandwickAustralia

**Keywords:** age‐related macular degeneration, chair‐side reference, drusen, infrared, retina

## Abstract

**Purpose:**

The purpose of this article is to describe the appearance of age‐related macular degeneration (AMD) phenotypes using infrared (IR) reflectance imaging. IR reflectance imaging of the retina has the potential to highlight specific sub‐retinal features and pathology. However, its role in macular disease, specifically AMD, is often underestimated and requires clarification.

**Recent findings:**

Recent advances in clinical methods, imaging and scientific knowledge may be integrated to improve the accuracy of disease stratification in AMD. In particular, IR imaging holds an underutilised sensitivity to detect reticular pseudodrusen, which have been repeatedly described as a high‐risk sign for late AMD.

**Summary:**

This article provides clinically relevant descriptions of AMD phenotypes using IR reflectance imaging. The findings are integrated with images from cases seen at the Centre for Eye Health. As primary eye‐care providers assume a critical role in the detection, diagnosis and management of AMD, we also provide a chair‐side reference to assist clinicians in interpreting IR images in AMD.

## Introduction

Age‐related macular degeneration (AMD) is a leading cause of blindness and visual impairment worldwide.^1^, ^2^ Early and intermediate AMD are typically asymptomatic and may be detected via careful, routine eye examination using multiple imaging methods. However, the effective use of advanced imaging in the clinical evaluation and management of AMD is presently difficult for several reasons: (1) AMD is heterogeneous – the presentation is variable and various phenotypes exist, (2) the pathophysiology of the condition is complex, multifactorial and not well understood, which limits the interpretation of clinical signs, (3) clinical signs typically associated with AMD may be due to other macular disorders, (4) advanced imaging modalities have been rapidly adopted by eye‐care professionals in general practice and no practical, standardised testing paradigm or widely disseminated clinical guidelines regarding the interpretation of advanced imaging in AMD exist. While use of these technologies can increase the likelihood of patients being recognised and treated as early as possible, it could potentially lead to an escalation of false positives and false negatives, as has been demonstrated in glaucoma.^3^

Late or advanced AMD may cause permanent central vision loss and disability due to either geographic atrophy or exudative changes (neovascular AMD). Treatment options are only available for the neovascular form of AMD although numerous other treatment options for AMD are currently being explored.^4^ Additionally, nutritional supplementation can reduce the risk of progression in patients with intermediate AMD.^5^ Therefore, clinical staging of the condition dictates the management plan and is important for eye‐care professionals when assessing AMD. Preventative strategies for AMD that maintain macular health require the accurate identification of early disease states. The identification of risk factors for progression to advanced stages is also important^6^ and eye‐care professionals worldwide have a duty of care to detect neovascular AMD early. Primary eye‐care providers are often the first point of contact for individuals presenting with reduced vision. They play a critical role in the detection and monitoring of AMD and timely referral to sub‐specialist services for treatment.

Traditional clinical characterisations of AMD were mainly obtained with ophthalmoscopy or fundus photography. The neovascular form was detected and classified additionally with fluorescein angiography, which can detect leakage.^7^ The latter technique has shown significant intra‐ and inter‐observer variability^8^ and its invasive nature precludes its use as a screening procedure. The last two decades has seen the simultaneous emergence of multiple advanced technologies for imaging and visual function measurement into clinical practice including optical coherence tomography (OCT), scanning laser ophthalmoscopy (SLO), infrared (IR) reflectance imaging and fundus autofluorescence. These imaging modalities allow for a variety of approaches to investigate the macula in health and disease and may be more precise than traditional methods at detecting morphological changes in early disease. The imaging modalities provide a structural image against which perimetry and other visual function targets are detected. Advanced ocular imaging can be applied in AMD for several reasons: (1) to detect ocular disease at an earlier stage, (2) to stratify phenotypes consistently in order to identify patients with or at risk of visual impairment, (3) to aid in distinguishing AMD from other macular disorders, (4) to evaluate for disease progression, and (5) to measure the response to treatment.

IR reflectance imaging (also described as near‐infrared imaging) has the potential to highlight sub‐retinal features and pathology by penetrating further through the fundus than other modalities.^9^ However, its role in the detection of macular disease is likely to be underestimated^10^ and requires clarification. The purpose of this article is to describe the appearance of AMD phenotypes using IR reflectance imaging. We systematically reviewed the literature focussing on the diagnostic utility of the technique, and subsequently collated and synthesised current clinically relevant descriptions of AMD phenotypes using IR imaging. We also provide a summary chair‐side reference to assist the clinician in interpreting IR images.

## Methods

### Literature review

A review of the English literature was performed by searching the National Institute of Health's PubMed database using the following combination of keywords and their relevant truncations and abbreviations: age‐related macular degeneration, age‐related maculopathy and infra‐red. The bibliographies from clinical guidelines on AMD^11^, ^12^, ^13^, ^14^ were also consulted for relevant material. Only seven articles were identifiable through current clinical guidelines. Articles that related to treatment efficacy, diseases other than AMD or imaging techniques outside of those described in the next section, were outside the scope of this review and specifically excluded. The full texts of 30 articles were included in the final analysis; articles were primarily retrospective and observational. Three articles enrolled a control group.^9^, ^15^, ^16^

### Principles of IR imaging

For the acquisition of IR fundus images, an IR light source is typically teamed with direct, confocal SLO to scan the retina sequentially in either a point‐by‐point or line‐by‐line manner.^17^ The intensity of the reflected light is measured at each point with a light‐sensitive detector, and subsequently used to generate an *en face* image.^18^ In this direct (bright‐field) mode of imaging, the size of the aperture (for instance a confocal aperture that is 200–800 μm with respect to the retina) governs the extent to which light scattered from layers other than the point of illumination is blocked. This property facilitates discrete z‐axis imaging and thus the separation of individual layers.^18^

IR imaging using confocal SLO can also be applied in an indirect (dark‐field) retro‐mode, whereby in a point‐by‐point scanning instrument a central occluder is introduced to form an annular aperture.^19^ Directly reflected light is blocked while scattered light passing through the outer annulus is returned to the detector.^20^ This technique has shown high sensitivity for the imaging of drusen.^21^ Tomographical imaging, as a series of 32 sequential images, using an aperture size of 24 μm diameter capable of providing measurements of retinal surface height has also been described with reference to AMD related macular cysts and pigment epithelial detachment.^22^, ^23^ Images obtained using any of these methods can be performed rapidly and non‐invasively without intravenous dye injection or pupil dilation.^9^, ^21^, ^24^

The human ocular fundus contains a variety of substances that variably absorb, reflect or scatter infrared light.^9^ The primary fundus molecules that absorb infrared light include: oxygenated haemoglobin, haemoglobin and water.^9^ In contrast, melanin is a strong reflector, even in near IR light, and has less absorption than in visible wavelengths.^16^ Macular pigment has a minimal effect.^9^ Any macular pathology characterised by sub‐cellular deposition of molecules and/or rearrangement of existing tissue may result in irregular effects on IR light scatter, reflection and absorption and consequently appear as greyscale contrast variations in IR images. Reflectance spectra in the IR range (>800 nm) appear independent of intra‐individual differences in pigmentation.^9^ IR imaging may also detect pathological changes despite the presence of haemorrhage or cataract.^9^ A key strength of IR imaging lies in its ability to visualise subretinal features that may be otherwise obscured or ambiguous using more traditional methods, such as retinal photography.^15^ Visualisation of such subretinal changes is maximised at wavelengths longer than 820 nm.^9^

### Technical specifications of IR imaging devices

Images acquired using direct, confocal SLO represent the primary standard of contemporary IR reflectance imaging, which is reflected in this manuscript's results and discussion. Most articles considered in this review used Spectralis Heidelberg Retina Angiograph 2 confocal SLO (Spectralis HRA2, Heidelberg Engineering, http://www.heidelbergengineering.com, λ = 815 nm, 30 × 30 degree field of view, 512 × 512 pixels minimum image size) for the acquisition of images. The HRA2 provides lateral optical and digital resolutions of 14 and 11 μm per pixel respectively. Used in high resolution mode, the lateral digital resolution is improved to 5 μm per pixel.^24^ Earlier, seminal works^9^, ^15^, ^21^, ^25^, ^26^ used a research prototype SLO (Ti:Sapphire Schwartz Electro‐optics, tuneable λ = 795–895 nm, maximum 29 × 23 degree field of view, 512 × 512pixels image size). Other lesser quoted devices include the: Topographic scanning system^22^, ^23^ (TopSS, Laser Diagnostic Technologies, http://www.zeiss.com/meditec/en_de/home.html, λ = 790 nm, capable of 10 × 10, 15 × 15 or 20 × 20 degree fields of view, 256 × 256 pixels image size), clinical SLO^15^ (Rodenstock, http://www.rodenstock.com, λ = 790 nm) and the F‐10 confocal SLO^27^ (Nidek Co, https://www.nidek-intl.com, λ = 790 nm).

Alternatives to SLO that utilise IR imaging are also available. For instance, IR illumination is also frequently used in the observation system of non‐mydriatic fundus cameras to guide image alignment during acquisition while avoiding pupillary constriction. However, due to the scatter of IR light over longer distances, IR imaging in this context is only able to reveal large or heavily pigmented structures.^9^ IR wavelengths have also been applied using GDx scanning laser polarimetry (Carl Zeiss Meditec, http://www.zeiss.com/meditec/en_de/home.html, λ = 780 nm, 15 × 15 degree field of view) to investigate different components of neovascular AMD.^28^

### Case images

IR images provided in this review for illustrative purposes were obtained by a retrospective review of noteworthy cases that presented between 2010 and 2015 to the Centre for Eye Health (CFEH) Sydney, Australia. IR images were acquired at CFEH as part of a standardised clinical testing protocol^29^ using the Spectralis HRA2, a 30 × 30 degree field of view centred on the macula and a minimum image resolution of 768 × 768 pixels. Retinal photographs were acquired using the Kowa 3D nonmydriatic retinal camera (Kowa, http://www.kowa.com). All patient records and images were independently reviewed by at least two experienced clinicians in concordance with CFEH's protocols.^29^ Final images presented herein were all reviewed by an ophthalmologist with a medical retina subspecialty (NA). Patient written consent was obtained in accordance with the Declaration of Helsinki and approved by the Biomedical Human Research Ethics Advisory Panel of the University of New South Wales, Australia.

## Results

### General appearance of the IR images of healthy and diseased retinas

**Figure 1:**
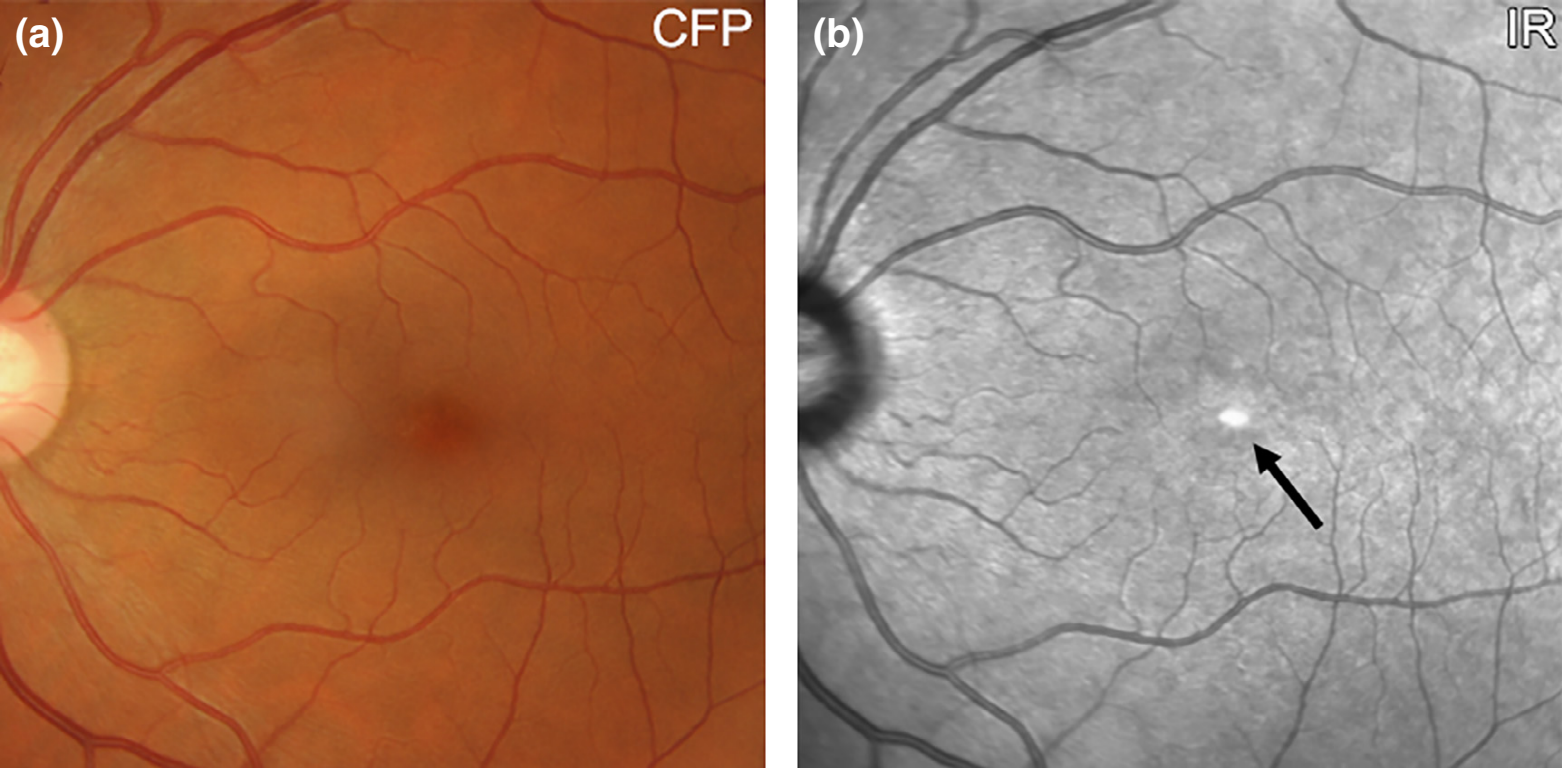
(a) Retinal photograph and (b) IR reflectance image of a 57 year old Caucasian female without any macular disease. The normal foveal ‘umbo’ appeared as a discrete, plaque‐like, hyper‐reflective spot in the IR image while the surrounding macula showed homogeneous reflectivity (arrow). Increased central IR signal may represent a reflection artefact or be attributable to the higher melanin content of foveal retinal pigment epithelium cells relative to more eccentric RPE. Choroidal and retinal vessels appear as dark shadows in the IR image. CFP, colour fundus photograph; IR, Infrared image.

IR imaging displays the normal foveal ‘umbo’ as a discrete, plaque‐like, hyper‐reflective spot using IR reflectance imaging, especially in young eyes.^30^, ^31^, ^32^ The surrounding macula typically shows homogeneous reflectivity with a slight decrease in reflectivity approaching the fovea (*Figure* 1).^16^ Increased central IR signal may represent a reflection artefact^24^ or may be attributable to the foveal slope^30^ and/or higher melanin content of foveal retinal pigment epithelium (RPE) cells relative to more eccentric RPE.^32^ Due to the absorption of light by blood, choroidal and retinal vessels are typically visualised in the normal eye and appear dark against a lighter fundus background. Retinal vessels are more clearly visualised than choroidal counterparts and may also feature dark borders.^9^

**Figure 2:**
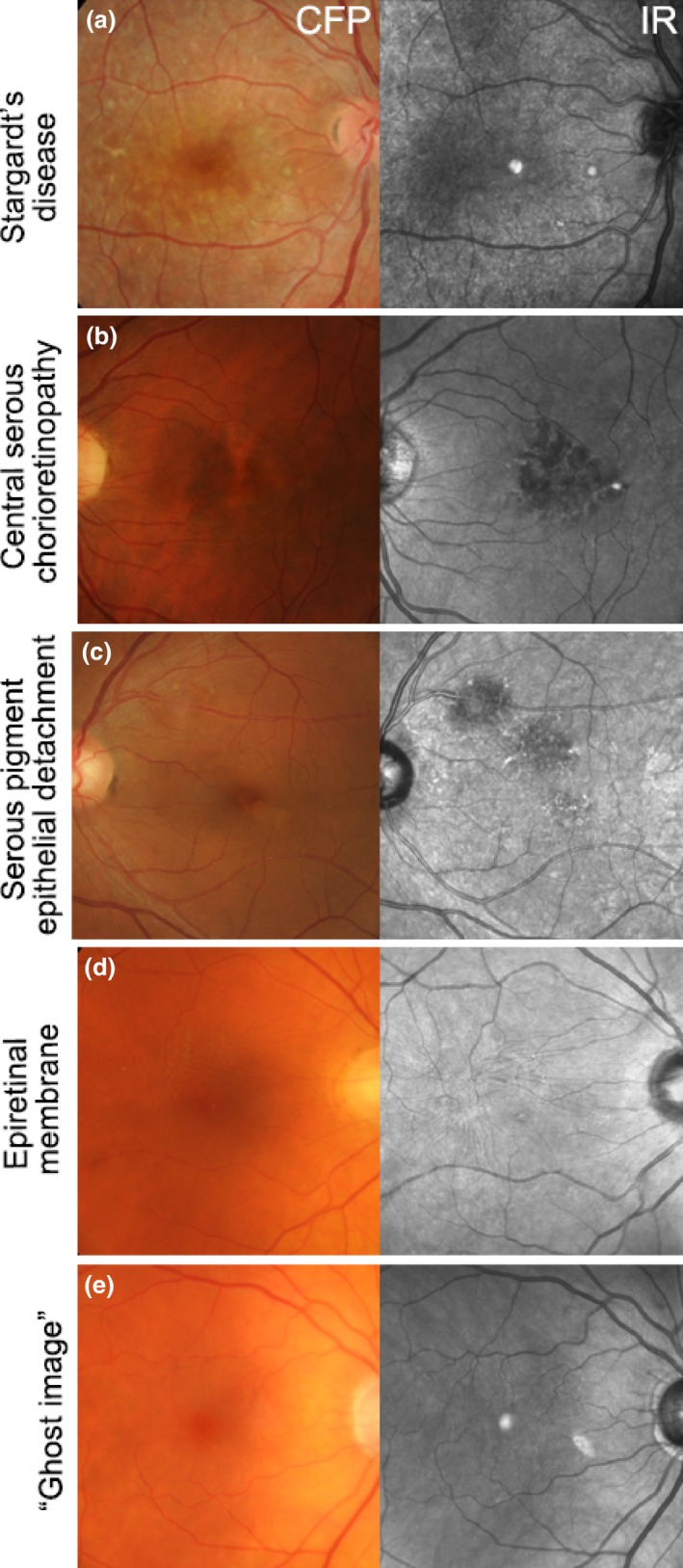
Using IR imaging, macular lesions can appear hyper‐reflective as in (a) Stargardt's disease, or hypo‐reflective as in (b) Central serous chorioretinopathy, (c) Serous pigment epithelial detachment and (d) Epiretinal membrane. (e) Ghost image in an eye with a posterior chamber intraocular lens. CFP, colour fundus photograph; IR, Infrared image.

Abnormalities on IR imaging may be classified as increased (hyper‐reflective; *Figure* 2 *a*), reduced or absent (hypo‐reflective; *Figure* 2 *b*–*d*).^32^ Macular lesions that may appear hyper‐reflective include: geographic atrophy or choroidal neovascularisation (CNV) of AMD and punctate atrophy in multifocal choroiditis or punctate inner choroidopathy.^30^ Conditions that show IR hypo‐reflectivity include central serous chorioretinopathy, serous pigment epithelial detachment^16^ and epiretinal membrane (*Figure* 2 *b*–*d*). ‘Ghost images’ (*Figure* 2 *e*), may also be observed and are a common artefact that mimics the hyper‐reflectivity of the foveal umbo. This artefact is likely to appear consistent between visits, be absent using fundus photography and fundus autofluorescence imaging, and can not be eliminated without reducing image quality.^30^ Ghost images show large intra‐individual variability in size, shape (circular, oval or crescent‐shaped), location (usually nasal or superonasal to the fovea) and reflectivity (varying from solid, bright white to a granular or speckled appearance) with well‐circumscribed or indistinct borders. Ghost images typically occur in the presence of a posterior chamber intraocular lens and have been attributed to light reflection and scatter off the lens.^30^ Its appearance in the individual may depend on variables such as retinal curvature, angle of incident light, lens tilt or degree of posterior capsular opacification.^30^ Overall, articles indicated that best‐practice interpretation of IR imaging results and the differential diagnosis of AMD from other macular disorders requires close examination, clinician acumen and awareness of IR artefacts. The evidence specifically supports the use of IR reflectance and combined imaging with a high resolution OCT system for closer assessment of pixel‐to‐pixel corresponding morphological changes.^24^, ^33^

### IR imaging of early to intermediate AMD

AMD is a phenotypically heterogeneous disorder unified by progressive degeneration in the photoreceptor, retinal pigment epithelium (RPE), Bruch's membrane and choriocapillaris layers of the macula. The Beckman initiative for macular research classification committee^34^ recently classified AMD into three stages: early, intermediate and late. Using that consensus, early AMD is defined by the presence of medium drusen only within two disc diameters of the fovea and intermediate AMD as having large drusen and/or any pigmentary abnormalities in the same area of interest.

The dichotomy between early and intermediate AMD highlights the clinical importance of being able to detect drusen of various sizes and associated pigmentary abnormalities. Reticular pseudodrusen are known to confer risk for AMD progression independently from drusen. Thus, patients with early or intermediate AMD should also be carefully examined for these lesions.^6^, ^35^, ^36^, ^37^, ^38^, ^39^ These three signs may also be observed in both stages of late AMD: geographic atrophy and neovascular (exudative) AMD. The following sections describe the application of IR imaging in observing these three signs.

#### Drusen

Drusen are focal extracellular deposits, principally composed of lipid, that form between the basal lamina of the RPE and the inner collagenous layer of Bruch's membrane.^40^ The term now applies to a diverse group that includes hard, soft and cuticular drusen, as well as reticular pseudodrusen.^41^, ^42^ Hard drusen appear as punctate, small‐medium sized (<125 μm), yellow‐white lesions with discrete borders.^35^ They are excluded as a defining sign of AMD if small (<63 μm)^43^ and may, in such instances, be described as normal aging changes.^34^ Soft drusen typically appear larger (63–≥1000 μm diameter) and less distinct.^35^, ^41^ They represent a risk factor for AMD progression to advanced stages.^44^ Cuticular drusen refer to numerous, clustered or radially distributed drusen and have been described as a phenotype of AMD that features an earlier age of onset (third decade).^42^, ^45^ Reticular pseudodrusen will be described further in the next sub‐section.

**Figure 3:**
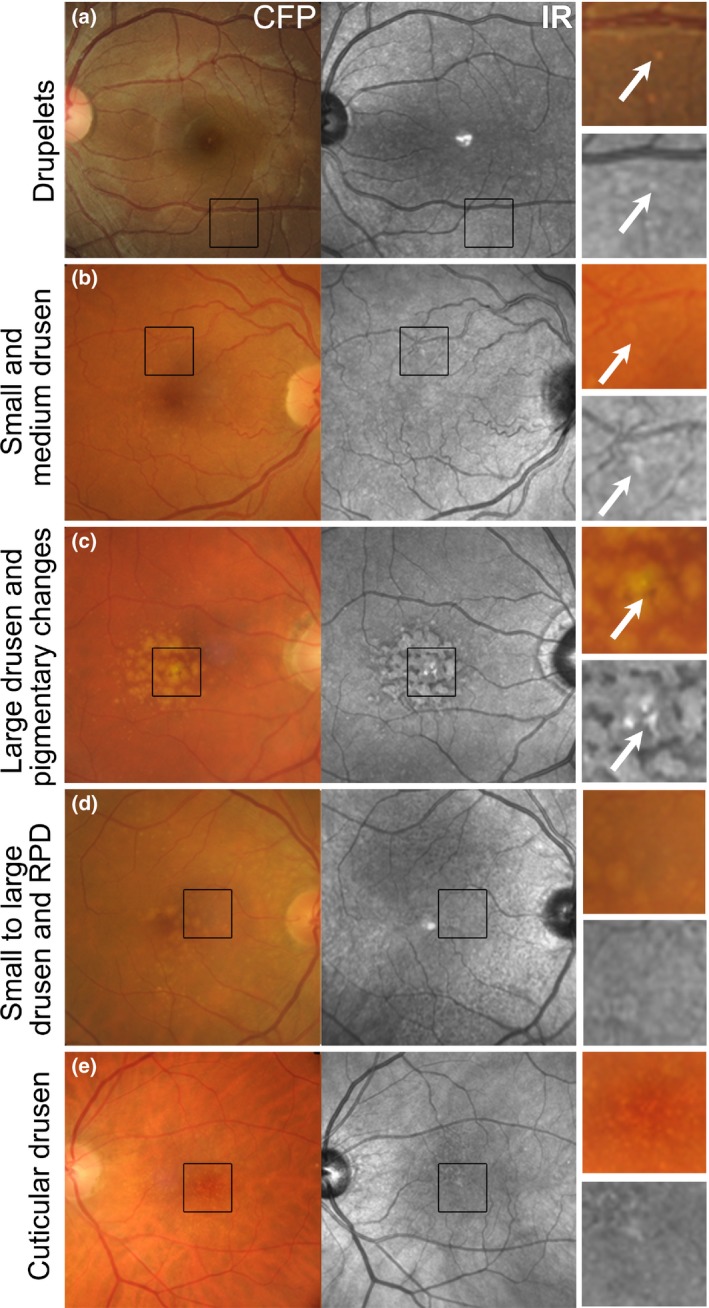
The appearance of drusen subtypes and pigment changes using IR imaging. (a) Drupelets (small drusen) present in a 29‐year‐old Caucasian female. (b) Early AMD characterised by small and medium sized drusen in a 69‐year‐old Caucasian female. The small and medium sized drusen in both cases appeared as well‐defined, disseminated spots of increased IR reflectivity. (c) Intermediate AMD in a 72‐year‐old Caucasian male with predominantly large drusen and central hyper‐pigmentary changes. The large drusen appear relatively well‐defined, mildly hyper‐reflective in comparison to the surrounds and are surrounded by hypo‐reflectivity in the IR image. Pigmentary changes appeared discrete and showed more intense hyper‐reflectivity than the drusen. (d) Intermediate AMD in a 73‐year‐old Caucasian female. The retinal photograph shows drusen ranging in size from small to large, predominantly affecting the central macula associated with variations in the greyscale tone of the IR image. Reticular pseudodrusen are also present and appear as grouped, hypo‐reflective spots most noticeable at the superior macula using the IR reflectance image. (e) Left eye images of a 65‐year‐old Caucasian female with cuticular drusen. The cuticular drusen appear punctate, hypo‐ and hyper‐reflective, and less distinct using IR imaging than in the CFP. CFP, colour fundus photograph; IR, Infrared image; RPD, reticular pseudodrusen.

Using IR imaging, drusen appear as well‐defined, disseminated, spotted areas of either increased or decreased reflectivity on IR imaging (*Figures* 3 *a*–*e and* 4 *a*)^16^, ^32^ and may preclude the visibility of posterior choroidal detail.^9^ Small and medium sized drusen (<125 μm) are more likely to appear hyper‐reflective (*Figure* 3 *a*,*b*), while the effect of large drusen (≥125 μm) is more inconsistent (showing either increased or reduced reflectivity; *Figures* 3 *c*,*d and* 4 *a*).^32^ These drusen related variations in greyscale tone on the IR image are classically subtle, and appear with low contrast.^41^ As a corollary, drusenoid pigment epithelial detachments (PEDs) adopt a patchy hyper‐/hypo‐pattern in IR imaging.^32^ The variable levels of IR reflectance seen with drusen (*Figure* 3 *d*) appears to be associated with pathological alterations in RPE thickness^32^, ^46^ and associated changes in the reflection profile of directional light off the fundus.^16^

**Figure 4:**
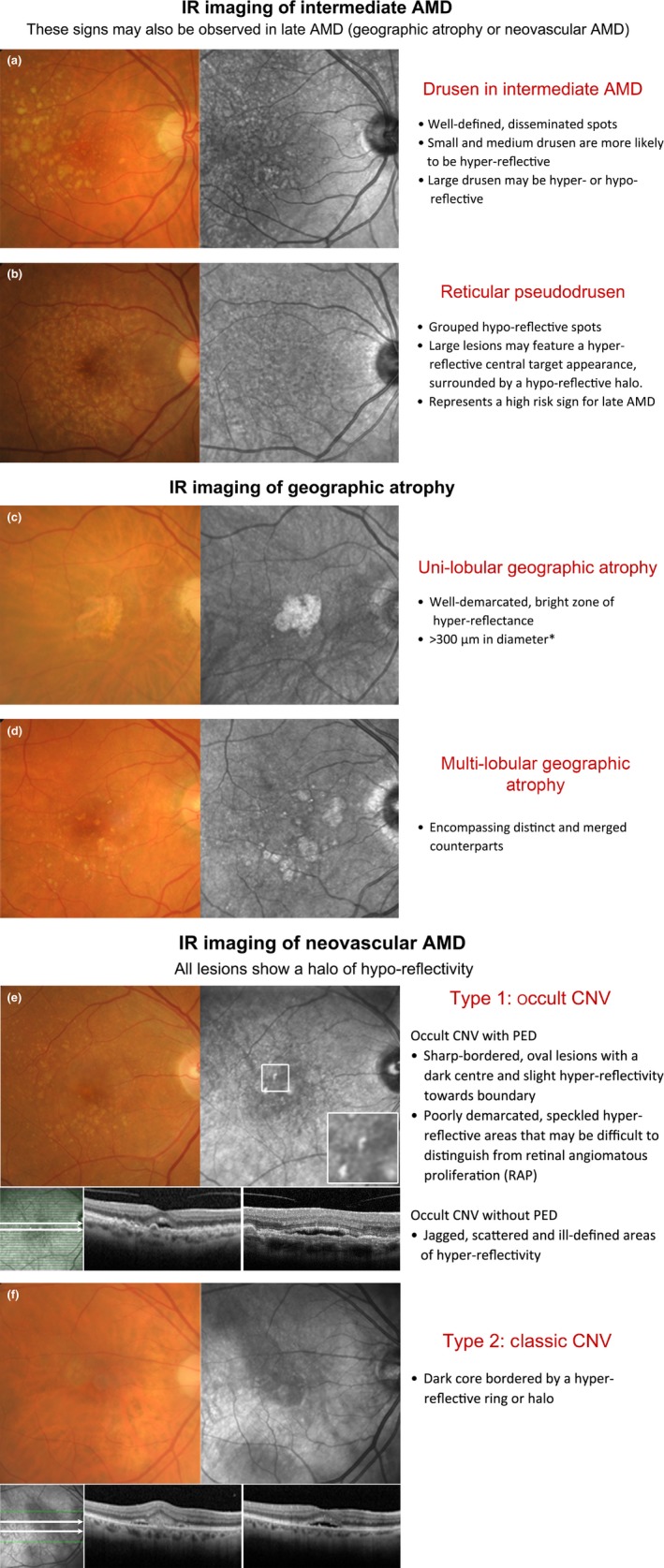
Double‐sided chair side reference chart designed to aid in the differential diagnosis of AMD phenotypes using IR imaging: (a) Intermediate AMD and scattered drusen of various sizes. The small and medium sized drusen (<125 μm) were predominantly hyper‐reflective, while the effect of large drusen (≥125 μm) was more inconsistent (both hyper‐ and hypo‐reflective). (b) Reticular pseudodrusen (RPD), which typically present as a reticular pattern of small, yellow‐white, round lesions using colour retinal photography and as groups of hypo‐reflective lesions using IR imaging. Larger lesions may be accompanied by a halo‐like appearance, also known as a ‘target’ aspect, with increased IR centrally surrounded by a halo of decreased reflectivity. (c,d) Geographic atrophy secondary to AMD; the atrophic regions appear as well‐demarcated, bright zones of hyper‐reflectivity, which relates to the absence of RPE. The presentation may be (c) uni‐lobular involving the fovea or (d) multi‐lobular, extrafoveal and/or associated with drusen of variable sizes. Note that the atrophic areas may be more apparent using IR imaging than retinal photography. (e,f) Neovascular AMD; the IR images reveal (e) sharp‐bordered, oval lesions and speckled hyper‐reflectivity that suggest occult CNV, and (f) a dark core bordered by a hyper‐reflective ring or halo consistent with the typical appearance in classic CNV. Cases were chosen based on the consistency of the IR image to previous descriptions provided in the literature. *A size criterion of 300 μm was adopted by Xu *et al*.^38^ Historical characterisations of atrophic lesions in AMD 35 applied a 175 μm cut off to retinal photography observing the difficulties in detecting choroidal vessels and edges of atrophic areas in smaller regions.

Cuticular (or basal laminar) drusen (*Figure* 3 *e*) appear less distinct in IR images than colour fundus photographs.^41^ They appear uniformly round, small‐medium (50–75 μm) in diameter and present in multiple, dense clusters reminiscent of a ‘starry‐sky’ using fluorescein angiography.^41^ Cuticular drusen resemble blunted triangles on cross section and feature greater RPE thinning at its apex than at its base, which causes local variations in the reflection and transmission of IR light.^41^

#### Reticular pseudodrusen (RPD)

RPD (also known as subretinal drusenoid deposits) have been described as a high risk sign for late AMD, with an estimated prevalence of 9–36% in AMD patients.^6^, ^35^, ^36^, ^37^, ^38^, ^39^ The high association between RPD and late AMD suggests that the former may represent a hallmark of the latter disease. Conversely, their absence may indicate the presence of other diseases that mimic the late AMD phenotype.^47^ Consequently, several articles^6^, ^31^, ^37^, ^38^, ^39^, ^47^, ^48^, ^49^, ^50^, ^51^, ^52^ specifically supported the inclusion of IR imaging in AMD assessment due to its high sensitivity for the detection of RPD. Discrimination of these lesions from other drusen in AMD is important because it allows the managing eye‐care professional to more accurately stratify the individual's risk for progression.^41^

RPD appear as a reticular pattern of small, yellow‐white, round or oval lesions using funduscopy and colour retinal photography, most frequently located in the superior outer macula and superotemporal to the macula (*Figures* 3 *d and* 4 *b*). In IR imaging, RPD classically appear as groups of hypo‐reflective lesions.^39^, ^49^, ^50^ Larger lesions may be accompanied by a halo‐like appearance, also known as a ‘target’ aspect, with increased IR centrally surrounded by a halo of decreased reflectivity.^31^, ^47^, ^50^, ^53^ The reasons for IR reflectivity of RPD remain unresolved.^37^ Querques *et al*.^53^ speculated that visualisation of the core may be explained by further heterogeneity in drusen type or formation, or may only occur if the image is focused on the centre of the drusen base.

Spaide^37^ recently relied on a diagnosis of RPD using IR SLO imaging only to provide a more unified description of RPD: lesions appearing as isolated, darker spots, some with a brighter central target appearance. In some eyes, the RPD appeared to be distributed in a fairly uniform fashion. In others, they appeared more amorphous, interconnected, ill‐defined and partially confluent, producing ribbon‐like patterns. He observed that RPD near the central macula tended to be smaller and more discrete compared to those further from the macula, which appeared more confluent and variable in terms of size and spacing.

Two studies have iterated the importance of distinguishing RPD from soft drusen.^38^, ^52^ The former classically appear grouped and hypo‐reflective while the latter tend to present as hyper‐reflective bodies scattered through the macula with variable shape and size.^38^ However, because both soft and hard drusen may occasionally present with hyporeflectivity in IR images,^16^ the clinician should be aware that specificity for the detection of RPD may be consequently reduced.

Compared with other modalities that are prone to signal attenuation by macular pigment, IR imaging may be especially necessary for detecting RPD in the central 1 mm of the macula, as well as detecting it in more subfields (i.e. showing a greater area of fundus involvement).^31^, ^39^, ^47^, ^48^, ^49^, ^52^ However, the changes are often subtle and identification requires careful analysis using images of good quality with even illumination, accurate centration and sharp focus over the entire frame, which may be challenging to acquire in all patients. Analysis may be hampered by the presence of other phenotypic characteristics.^50^

Finger *et al*.^6^ emphasised that the combination of both spectral‐domain OCT (SD‐OCT) and IR imaging allows the detection of RPD with the highest possible accuracy. These authors reported a 58% prevalence of RPD in the fellow eye of patients with unilateral CNV using their combined imaging protocol; the presence of RPD was defined as ‘groups of hypo‐reflective lesions against a background of mild hyper‐reflectance on IR with corresponding hyper‐reflective signal above the retinal pigment epithelium on SD‐OCT’. Others^38^ have reported that 93% (114/123) of eyes with primary GA due to AMD exhibited reticular macular disease when the area criterion for the reticular pattern was required to span at least two contiguous disc areas in at least one imaging modality (IR or fundus autofluorescence confocal SLO images). These discrepancies in reported prevalence of RPD are likely due to the imaging modalities used in their detection.^38^, ^39^, ^47^, ^48^

Ueda‐Arakawa *et al*.^51^, ^52^ also recommended using at least two imaging modalities for a more accurate diagnosis of RPD. They systematically compared the sensitivities, specificities and agreement rates regarding RPD using different imaging modalities (the blue channel of color fundus photography, IR, fundus autofluorescence, near‐infrared fundus autofluorescence, confocal blue reflectance indocyanine green angiography and SD‐OCT).^52^ IR imaging showed an agreement rate of 91% and featured the highest sensitivity (95%) and false positive rate (8%; 15/183). It also had the lowest specificity for the detection of RPD at 92% in contrast to the other techniques (which ranged from 95 to 100%). IR and SD‐OCT may be most useful for screening of RPD (due to their high sensitivity) while another modality with 100% specificity (such as the blue channel of color fundus photography) may be useful for confirmation of the phenotype.^52^ Other groups have corroborated these findings showing a high prevalence of RPD^47^, ^48^, ^50^ and similarly good inter‐observer agreement^47^, ^50^ using IR imaging.

#### Pigmentary changes

Pigmentary abnormalities occurring within two disc diameters of the macular centre carry a strong association with the subsequent development of late AMD and should be identified in all AMD patients.^34^ The IR appearance of pigmentary changes in AMD varies between discrete, clumped deposits of intense hyper‐reflectivity (*Figure* 3 *c*)^23^, ^54^ and hypo‐reflectivity.^15^ This likely relates to the non‐uniform deposition of melanin and other compounds, such as lipofuscinoid fluorophores and pigmented melanocytes.^54^ Pigmentary changes that appear hyper‐reflective on IR imaging likely feature higher levels of melanin relative to surrounding retina.

### IR imaging of late AMD

Late or advanced AMD may cause permanent central vision loss and disability due to either geographic atrophy or exudative changes (neovascular AMD). Left untreated, neovascular AMD causes severe visual impairment whereas the vision loss related to geographic atrophy tends to show a more gradual disease course.^55^

#### Geographic atrophy (GA)

GA represents the hallmark of non‐neovascular late AMD, and is characterised by confluent and progressive RPE cell death and attenuation of the choriocapillaris, Bruch's membrane and photoreceptor layer. It may be described as ‘foveal sparing’ or ‘foveal involving’ and typically forms as distinct parafoveal patches that coalesce over time.^56^

IR imaging of GA appears as well‐demarcated, bright zones of hyper‐reflectance spanning at least 300 μm in diameter (*Figure* 4 *c*,*d*).^32^, ^33^, ^38^ The areas may be uni‐ or multi‐lobular (also known as multifocal, encompassing both distinct and merged counterparts) depending on the number of visible areas.^38^ Interestingly, Xu *et al*.^38^ showed that GA lesions which appeared as single, large, central lesions in IR imaging tended to appear as condensations of densely packed smaller lobules using fundus autofluorescence concluding that the majority of GA related to AMD is multi‐lobular. The hyper‐reflective appearance of GA in IR images relates to the absence of RPE blockage^32^ (compared to neighbouring regions) and reflection of light from the sclera.^57^

A novel sign termed ‘ghost drusen’ in AMD‐related geographic atrophy has been further characterised using IR imaging.^58^ The lesions appear as hyper‐reflective pyramidal structures using SD‐OCT and as ‘hypo‐reflective lesions surrounded by hyper‐reflective halos’ within the background hyper‐reflectivity that characterises the encompassing GA area. Rarely, the lesions also featured discrete hyper‐reflective dots internally. These lesions were only described in one article.^58^

#### Neovascular AMD

In neovascular AMD, the development of choroidal neovascularisation (CNV) in the subretinal space leads to the exudation of fluid, blood and lipid and subsequent fibrous scarring. Thus, the neovascular form may be systematically characterised by the presence or absence of focal or widespread RPE dysfunction and detachment (serous, drusenoid, fibrovascular or haemorrhagic), subretinal haemorrhage or fluid, cystoid macular oedema, retinal haemorrhages, hard exudates, RPE tears and epiretinal or subretinal fibrous or glial scar.^35^

Fluorescein angiography has been historically recognised as the gold standard technique for diagnosis and follow up of neovascular AMD. CNV subtypes include: classic (type 2, well‐defined and sub‐retinal), occult (type 1, ill‐defined and underlying the RPE) and mixed (predominantly or minimally classic, showing combinations of classic and occult features). Another less common variant termed retinal angiomatous proliferation (RAP) features intraretinal neovascularisation that subsequently anastomoses with neovascularisation in the subretinal space.^55^, ^59^, ^60^ Historically, the angiographic distinction of subtypes has afforded high clinical significance, owing to links with treatment efficacy and the natural history of disease.^55^, ^59^, ^60^ Management guidelines^12^, ^61^ that also describe the prognosis of neovascular subtypes are readily available.

IR imaging allows the subretinal extent of CNV to be better visualised by penetrating deeper through retinal tissue than other wavelengths (*Figure* 4 *e,f*). This may lead to earlier detection,^21^ localisation and thus better management of CNV cases. IR imaging of classic CNV presents as a dark core bordered by a hyper‐reflective ring or halo.^9^, ^10^, ^16^, ^21^ The ring may appear as either closed (O shaped) or as a horseshoe (U‐shaped), and is likely to correspond with the borders of the neovascularisation.^10^ Occult CNV without pigment epithelial detachment (PED) has been described in the literature as ‘jagged’, scattered areas of hyper‐reflectivity with indeterminate borders in IR reflectance imaging.^16^ The central hypo‐reflective core may represent the vascular component of CNV^21^ and may also appear thickened or display associated tractional striae.^9^, ^21^ Cases with PED similarly do not readily transmit light,^9^ and are revealed as sharp‐bordered, oval lesions with a dark centre and slight hyper‐reflectivity towards its boundary. These findings are consistent with earlier studies, which highlighted PEDs as appearing darker than the surrounds in IR light.^23^ Bastian *et al*.^62^ suggested that increased reflectance signals in an area of AMD related PED may represent a negative prognostic marker as this was observed in 87% of cases prior to the development of an RPE tear. RAP lesions adopted a ‘speckled’ pattern of focal increased IR reflectance.^16^ They often present with PED or other signs, such as blot shaped intraretinal haemorrhages, microaneurysms or telangiectatic vessels.^25^

Alterations in the IR signal corresponding with areas of CNV may also be characterised independent of the angiographic characterisations using their inherent features as follows. Consistent with the descriptions above, the IR reflectance signal is predominantly reduced i.e. hypo‐reflective centrally in active CNV due to light absorption and scatter of extraneous protein‐rich fluid or blood.^9^, ^16^, ^21^, ^23^ Theelan *et al*.^16^ further described that the reactive proliferation of RPE (which contains IR‐reflecting melanin) likely leads to IR hyper‐reflectivity which may offset fluid associated absorption. Finally, the irregular shaped areas which characterise some subtypes appeared to relate to the irregular accumulation of fluid and the summation of several optical effects.^16^ Exudates and fibrin typically provide a marked refractive change and consequently display intense IR hyper‐reflectivity.^23^ The accumulation of fibrin characteristic of classic CNV may also explain the bright hyper‐reflective ring seen in IR images as fibrin may be a more significant source of IR reflectivity in neovascular AMD, relative to melanin.^10^, ^16^ Consequently, the scattered pattern of hyper‐reflectivity that occurs in occult CNV is consistent with histological observations of diffuse fibrin deposition.^16^ The interpretation regarding the pattern of reflectivity in RAP lesions was confounded because most RAP cases were associated with occult CNV.^16^

Cystoid macular oedema occurs in several conditions (including neovascular AMD) and appears using IR imaging as subtly detectable round to oval shaped structures of low contrast that may radiate out from the centre of the fovea.^26^ Pilotto *et al*.^27^ reported respective sensitivity and specificity scores of 25% and 89% for neuroretinal detachment, 50% and 69% for PED and 38% and 100% for cystoid macular oedema using IR imaging. Finally, fibrosis or scarring displays intense hyper‐reflectivity using IR reflectance imaging and often obscure underlying changes.^9^, ^23^

Overall, IR reflectance imaging using confocal SLO has been described as superior to colour fundus imaging for CNV detection.^28^ The distinction of neovascular lesion subtype and activity is possible using IR imaging because of intrinsic differences in the spectral reflectance properties of compounds that occur in this form of late AMD.

### Limitations of IR reflectance imaging

In its current commercially available form, IR reflectance imaging generates an *en face*, two‐dimensional, monochromatic image, restricted to a 30 × 30 degree field of view. Eye‐care professionals in practice however are more familiar with colour images,^18^ and therefore the monochromatic changes seen on IR images are often subtle^52^ when considered against the traditional ‘gold standards’ of funduscopy or retinal photography. In routine clinical practice, management decisions must be made in a timely manner and the practicing eye‐care professional may not always be present at the time of imaging or have immediate access to results while seeing patients. Quality images (evenly illuminated, accurately centred on the fovea, sharply focused) may also be difficult to acquire.^47^, ^50^ In neovascular AMD, absolute visualisation of the neovascular network is not possible and current stratification of this late AMD phenotype is still restricted to detection of indirect signs (fluid, blood, exudates). Additionally, IR reflectance images, in their most widely accessible form, cannot provide direct quantitative measures of retinal thickness or information in three dimensions. Restriction of the illumination wavelength to an IR band enables subretinal structures to be emphasised at the expense of other layers.^15^ Information regarding structures outside of the plane of focus must be inferred based on focus, motion parallax or shadow cues.^21^

The current day use of IR imaging appears primarily limited by its accessibility: the modality has been labelled as a ‘research tool’ and is not always available clinically.^39^ The benefits of SLO technology are well known though expensive; images such as those of this manuscript are only commercially available using one combination OCT system (Spectralis). Some of the other OCT systems use instead the summed voxel projection technique for the fundus reference.^63^ Consequently, IR imaging may be relegated as secondary or adjunctive to other more directly intuitive imaging techniques such as structural optical coherence tomography (*Figure* 4 *e*,*f*) and colour fundus photography. More likely still, a combination of imaging modalities (multimodal imaging) used in tandem will provide the best available evidence for the management of patients.

## Conclusion

AMD has several pathological changes that can be visualised by IR imaging, as described in *Table* 1. A chair‐side reference is given in *Figure* 4, and illustrations of potential misdiagnoses are also given. As a whole, the collective clinical application and experience with advanced retinal imaging methods is still relatively new^30^ and evolving. However, the evidence base revealed in our results outlines the clear capability of IR imaging to stratify AMD phenotypes, in particular RPD. Thus, practising clinicians likely to see AMD patients should keep RPD and the potential value of IR imaging top of mind when evaluating the individual's risk for progression to advanced AMD.

**Table 1:** Summary regarding the reasons for the appearance of AMD phenotypes using IR imaging

AMD phenotype	Appearance on IR imaging	Reasons for IR reflectivity
Drusen	Well‐defined, disseminated spots Hyper‐ or hypo‐reflective Small and medium drusen are more likely to appear hyper‐reflective Large drusen may appear either hyper‐ or hypo‐reflective	Alterations in RPE thickness and associated changes in the reflection profile of directional light off the fundus
Pigmentary changes	Discrete, clumped deposits of intense hyper‐ or hypo‐reflectivity	Melanin and other compounds
Reticular pseudodrusen	Grouped hypo‐reflective spots Larger lesions may feature a hyper‐reflective central target appearance, surrounded by a hypo‐reflective halo	Unresolved
Geographic atrophy	Well‐demarcated, bright zones of hyper‐reflectance Uni‐ or multi‐lobular	Absence of RPE blockage (compared to neighboring regions) and reflection of light from the sclera
Neovascular AMD	All lesions show a halo of hypo‐reflectivity Classic CNV Dark core bordered by a hyper‐reflective ring or halo Occult CNV without PED Jagged, scattered and ill‐defined areas of hyper‐reflectivity Occult CNV with PED Sharp‐bordered, oval lesions with a dark centre and slight hyper‐reflectivity towards its boundary Poorly demarcated speckled areas of hyper‐reflectivity that may be difficult to distinguish from the appearance of RAP RAP Speckled focal increase	Increased light absorption and scatter of extraneous protein‐rich fluid or blood in the fundus Accumulation of fibrin may also explain the bright hyper‐reflective ring seen in IR images of classic CNV and the scattered pattern of hyper‐reflectivity in occult CNV

Conversely, data relating to the application of IR imaging for earlier detection and differential diagnoses was sparse. The causes of IR reflectivity in both health and disease also appear still to be unconfirmed. Furthermore, most articles relied on a retrospective and observational study design, which highlights an inherent limitation of the literature. The longitudinal surveillance and treatment of AMD are beyond the scope of this article and were specifically excluded from our results.

There exists a significant burden of disease related to AMD worldwide and a clinical paradigm shift incorporating advanced imaging into primary eye‐care appears to be underway, as evidenced by the rapid uptake of such tools by eye‐care professionals. This evolving environment has created a clear demand for the dissemination of clinical guidelines on the interpretation of advanced imaging to practising health professionals. These research results should provide the foundation for subsequent in‐depth analysis of clinical predictors of AMD progression and will facilitate development of international teaching paradigms.

## Disclosure

The authors report no conflicts of interest and have no proprietary interest in any of the instruments mentioned in this article.
